# From Birds to Bacteria: Generalised Velocity Jump Processes with Resting States

**DOI:** 10.1007/s11538-015-0083-7

**Published:** 2015-06-10

**Authors:** Jake P. Taylor-King, E. Emiel van Loon, Gabriel Rosser, S. Jon Chapman

**Affiliations:** Mathematical Institute, University of Oxford, Andrew Wiles Building, Radcliffe Observatory Quarter, Woodstock Road, Oxford, OX2 6GG UK; Institute for Biodiversity and Ecosystem Dynamics, University of Amsterdam, Science Park 904, 1098 XH Amsterdam, The Netherlands; SpaceTimeLab, Department of Civil, Environmental and Geomatic Engineering, UCL, Gower Street, London, WC1E 6BT UK

**Keywords:** Velocity jump process, Transport equations, Brownian limit, Non-Markov correlated random walk, Mean squared displacement, Effective diffusion

## Abstract

There are various cases of animal movement where behaviour broadly switches between two modes of operation, corresponding to a long-distance movement state and a resting or local movement state. Here, a mathematical description of this process is formulated, adapted from Friedrich et al. (Phys Rev E, 74:041103, 2006b). The approach allows the specification *any* running or waiting time distribution along with any angular and speed distributions. The resulting system of integro-partial differential equations is tumultuous, and therefore, it is necessary to both simplify and derive summary statistics. An expression for the mean squared displacement is derived, which shows good agreement with experimental data from the bacterium *Escherichia coli* and the gull *Larus fuscus*. Finally, a large time diffusive approximation is considered via a Cattaneo approximation (Hillen in Discrete Continuous Dyn Syst Ser B, 5:299–318, 2003). This leads to the novel result that the effective diffusion constant is dependent on the mean and variance of the running time distribution but only on the mean of the waiting time distribution.

**Electronic supplementary material:**

The online version of this article (doi:10.1007/s11538-015-0083-7) contains supplementary material, which is available to authorized users.

## Introduction

In nature, organisms whose sizes differ by many orders of magnitude have been observed to switch between different modes of movement. For instance, the bacterium *Escherichia coli* changes the orientation of one or more of its flagella between clockwise and anticlockwise to achieve a *run-and-tumble*-like motion (Berg 1983, 1990). As a result, during the runs, we see migration-like movement, and during the tumbles, we see resting or local diffusion behaviour.^1^ To add to this complexity, it should be noted that the direction of successive runs is correlated. On a larger scale, one could consider migratory movements of vertebrates where individuals often travel large distances intermittent with stop overs to rest or forage. An example, used in this paper, is the lesser black-backed gull (*Larus fuscus*). Individuals of this species that breed in the Netherlands migrate southwards during Autumn. Even though the scales involved in these two processes differ by many orders of magnitude, one can use a similar mathematical framework to model the observed motion.

The use of mathematical models to describe the motion of a variety of biological organisms, including bumblebees (Kareiva and Shigesada 1983), plants (Cain 1990) and zebra (Brooks and Harris 2008), has been the subject of much research interest for several decades. Early approaches were predominantly centred on the position jump model of motion (Brenner et al. 1998; Skellam 1951), where agents undergo instantaneous changes of position according to a distribution kernel interspersed with waiting periods of stochastic length. The position jump framework suffers from the limitation that correlations in the direction of successive runs are difficult to capture; this directional persistence is present in many types of movement (Marsh and Jones 1988). Furthermore, the diffusive nature of the position jump framework results in an unbounded distribution of movement speeds between successive steps. A related framework that is more realistic for modelling the motion of organisms is the velocity jump (VJ) model (Othmer et al. 1988), in which organisms travel with a randomly distributed speed and angle for a finite duration before undergoing a stochastic reorientation event. The VJ process is also referred to in the literature as a continuous non-Markovian correlated random walk.

In most formulations of the VJ process, there is an assumption that events occur as a Poisson process, which is manifested as a constant rate parameter in the resulting differential equation. In the position jump framework, non-exponentially distributed wait times and non-Gaussian kernel processes have been formulated, leading to fractional diffusion equations (Klafter 1987; Metzler and Klafter 2000). Recently, it has become clear how to extend the VJ framework to allow for more general distributions of interest (Friedrich et al. 2006a, b).

In many VJ models, it is assumed that resting states are largely negligible (Erban and Othmer 2004, 2005); this can be attributed to a focus on organisms with only momentary resting states, which has the benefit of alleviating some mathematical complexity whilst not changing the result significantly (Erban and Othmer 2004). However, in the work by Othmer et al. (1988), Erban and Othmer (2007) and Hillen (2003), it was shown that resting states can be included and are sometimes required in order to obtain adequate fits to experimental data (Othmer et al. 1988). Our goal in this paper is to extend the work by Friedrich et al. (2006b) to incorporate stationary resting states of finite duration, drawn from an arbitrary probability distribution—which their model did not allow for—following the formalism of Othmer et al. (1988).

The mathematical complexity of Friedrich’s model is such that finding solutions analytically or numerically is, in general, impractical. In the original paper, simplifications were made, which led to a fractional Kramers–Fokker–Planck equation, which has a known analytic solution (Friedrich et al. 2006b). However, Friedrich’s model was postulated to describe non-Gaussian kinetics in a weakly damped system; in contrast, we are considering a system in which biological agents generate their own momentum, which is related to self-propelled particle models (for example, Hagen et al. 2011). In the absence of such obvious simplifications for our system, we instead exploit methods to extract summary statistics from the governing equations, which may in turn be compared with experimental data.

After presenting the model, we derive the mean squared displacement (MSD). Using high-quality data describing the movement of *E. coli* and *L. fuscus*, we show that the MSD for the model and experimental data aligns. Similar comparisons between Markovian correlated random walks and experimental data can be found here (Bovet and Benhamou 1988; Casellas et al. 2008; Gautrais et al. 2009). A novel aspect of our approach is that, provided the behaviour being modelled is well described by the run and stop modes of motion, the parameters can be extracted on a microscopic scale prior to any numerical solution, and then, macroscopic behaviour can be derived *without* optimising or trying to fit data *a posteriori*.

Since the dynamics of the experimental data and those of the generalised VJ model achieve a close match, we explore numerically tractable simplifications to the equations. Most notably, we investigate the Cattaneo approximation, following the work by Hillen (2003, 2004).

Finally, it should be noted that the model presented does not take into account interactions between biological agents or with the environment. Whilst such effects are beyond the scope of the current study, it should be possible to extend the theory to incorporate these phenomena. In particular, the VJ process has roots in kinetic theory, which describes attractive and repulsive forces between atoms; models have been developed for biological agents to act comparably (Carrillo et al. 2009; Degond et al. 2004; Naldi et al. 2010). Interactions between agents and the surrounding environment have also been modelled for static environments and for dynamic signalling via diffusing chemical gradients (Chauviere et al. 2010; Erban and Othmer 2004, 2005).

## Two-State Generalised Velocity Jump Process

Consider a biological agent that switches stochastically between running and resting behaviour. During a running phase, the organism travels with constant velocity; during a resting phase, it remains stationary. Upon resuming a run following a rest, a new velocity is selected randomly. This motion is governed by three primary stochastic effects. We specify these by probability density functions (pdfs), as given below.

1. Waiting time: The time spent during a resting phase, denoted $\omega$, is governed by the pdf $f_{\omega }(t)$, where $\int _0^\infty f_\omega (t)\text {d}t=1$.
2. Running time: The time spent during a running phase, denoted $\tau$, is governed by the pdf $f_{\tau }(t)$, where $\int _0^\infty f_\tau (t)\text {d}t=1$.
3. Reorientation: We allow velocities from one run to another to be correlated between rests. We denote the velocity during the running phase immediately before a rest by $\varvec{v}'$ and the velocity in the post-rest running phase by $\varvec{v}$, where $\varvec{v}',\varvec{v}\in V$, for velocity space $V\subset {\mathbb {R}}^n$ in *n* spatial dimensions. The velocity $\varvec{v}$ is dependent on $\varvec{v}'$ and is newly selected upon re-entering a running phase, governed by the joint pdf $T(\varvec{v},\varvec{v}')$. We assume that this reorientation pdf is separable, so that $T(\varvec{v},\varvec{v}')\,=\,g(\varvec{\theta }, \varvec{\theta }')h(s, s')/s^{n-1}$ where $\varvec{\theta }$ is a vector of length $(n-1)$ containing angles and $s\,=\,\left| \left| \varvec{v}\right| \right|$ is the speed. In two dimensions, the turning kernel is decomposed as follows:
  - The angle distribution: $g(\theta , \theta ')$ requires the normalisation $\int _0^{2\pi }g(\theta , \theta ')\text {d}\theta \,=\,1$.
  - The speed distribution: $h(s, s')$ requires the normalisation $\int _0^{\infty }h(s, s')\text {d}s = 1$.

To further reinforce the process we are describing, we give a simple Gillespie algorithm (Gillespie 1977) for generating a sample path up until time $T_{\text {end}} > 0$. It should be noted that the sample path will need to be truncated as the algorithm generates positions beyond $T_{\text {end}}$.
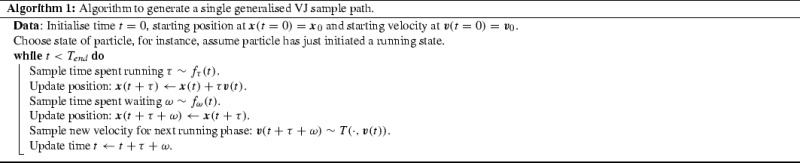


By considering the density of particles in a running state and the density of particles in a resting state, we can write down coupled differential equations for these states. The derivation is lengthy and similar in spirit to Friedrich et al. (2006b); hence, we provide only the main result here; full details are given in Appendix 1. We define $p=p(t,\varvec{x},\varvec{v})$ to be the density of particles at position $\varvec{x}\in \Omega \subset {\mathbb {R}}^n$, with velocity $\varvec{v}\in V\subset {\mathbb {R}}^n$ at time $t\in {\mathbb {R}}^+$ and $r\,=\,r(t,\varvec{x},\varvec{v})$, the density of those particles resting at $(t, \varvec{x})\in {\mathbb {R}}^+\times \Omega$, having just finished a jump of velocity $\varvec{v}\in V$. Note that this encodes an orientation to the resting state. Our analysis leads to the following equations

1 $$\begin{aligned} \left( \frac{\partial }{\partial t} + \varvec{v}\cdot {\nabla _{\varvec{x}}} \right) p(t,\varvec{x},\varvec{v})= & {} -\int _0^t \Phi _\tau (t-s) p(s,\varvec{x} - (t-s)\varvec{v},\varvec{v}) \text {d}s \\&+ \int _0^t \Phi _{\omega }(t-s)\int _V T(\varvec{v},\varvec{v}')r(s,\varvec{x},\varvec{v}')\text {d}\varvec{v}'\text {d}s, \nonumber \end{aligned}$$

and

2 $$\begin{aligned} \frac{\partial }{\partial t}r(t,\varvec{x},\varvec{v}) = -\int _0^t \Phi _\omega (t-s) r(s,\varvec{x},\varvec{v}) \text {d}s + \int _0^t \Phi _{\tau }(t-s)p(s,\varvec{x}-(t-s)\varvec{v},\varvec{v}) \text {d}s, \end{aligned}$$

where the delay kernels, $\Phi _i$ for $i = \tau , \omega$, are defined in Laplace space by

3 $$\begin{aligned} \bar{\Phi }_i (\lambda ) = \frac{\lambda \bar{f}_i (\lambda )}{1 - \bar{f}_i(\lambda )} \quad \text {for }i=\tau , \omega . \end{aligned}$$

where $\bar{f}_i$ is the Laplace transform of the pdf for the running and waiting time, respectively. When the waiting time is chosen as exponential,^2^ this is consistent with work by Othmer et al. (1988) and Rosser (2012).

Finding closed forms of $\Phi _i(t)$ is non-trivial for most choices of distribution $f_i(t)$. In Appendix 2, we examine the small time behaviour of $\Phi$ and identify the sizes of potential impulses at $t=0$. For the remaining non-singular behaviour, in the cases where we know the Laplace transform of $f_i(t)$, we then have an analytic expression for $\bar{\Phi }(\lambda )$, which can be inverted numerically using either a Talbot inversion or an Euler inversion (Abate 1995; Murli and Rizzardi 1990).

## Mean Squared Displacement

Equations (1–2) give us a system of delay integro-partial differential equations with $(2n + 1)$ degrees of freedom. With this level of complexity, a full analytic or numerical solution is impractical without first making simplifications. We therefore consider how to estimate the second spatial moment, i.e. the MSD (Othmer et al. 1988).

For the test function $\varphi = \varphi (\varvec{x}, \varvec{v})$ and arbitrary density $\rho = \rho (t,\varvec{x},\varvec{v})$,

5 $$\begin{aligned} Q_\rho (\varphi ,t) = \int _V \int _\Omega \varphi (\varvec{x}, \varvec{v})\rho (t,\varvec{x},\varvec{v}) \text {d}\varvec{x} \text {d}\varvec{v}. \end{aligned}$$

This gives the expected value of $\varphi$ over the space $V\times \Omega$ at time t, weighted by density $\rho$. By using test functions $\varphi = 1, \left| \left| \varvec{x}\right| \right| ^2, \varvec{v}\cdot \varvec{x}, \left| \left| \varvec{v}\right| \right| ^2$, we associate $N_\rho (t) = Q_\rho (1,t)$ as the number of particles in state $\rho$ and $D_\rho ^2 (t)= Q_\rho (\left| \left| \varvec{x}\right| \right| ^2, t), B_\rho (t) = Q_\rho (\varvec{v}\cdot \varvec{x}, t)$ and $V_\rho ^2 (t)= Q_\rho (\left| \left| \varvec{v}\right| \right| ^2, t)$ as the mean squared displacement, the mean velocity-displacement and the mean squared velocity weighted by $\rho$, respectively. We can then obtain a closed system of integro-differential equations for these quantities. In order to make progress, we must first make some assumptions on the turning kernel *T*. By considering that the mean post-turn velocity has the same orientation as the previous velocity, we define the index of persistence $\psi _d$ via the relation

6 $$\begin{aligned} \bar{\varvec{v}}(\varvec{v}') = \int _V \varvec{v}T(\varvec{v},\varvec{v}') \text {d}\varvec{v} = \psi _d \varvec{v}'. \end{aligned}$$

Informally, this means that turning angles between consecutive velocities have zero mean. We also require that the average mean squared speed is a constant

7 $$\begin{aligned} S_T^2(\varvec{v}') = S_T^2 = \int _V \left| \left| \varvec{v}\right| \right| ^2 T(\varvec{v},\varvec{v}') \text {d}\varvec{v} , \end{aligned}$$

this corresponds to a memoryless turning kernel in speed, i.e. $h(s,s') = h(s)$. Finally, for unconstrained motion where $\Omega = {\mathbb {R}}^n$, we see that delays in space correspond to inclusion of other moments, that is,

8 $$\begin{aligned} \int _V \int _\Omega \left| \left| \varvec{x}\right| \right| ^2 \rho (t,\varvec{x}-c\varvec{v},\varvec{v}) \text {d}\varvec{x} \text {d}\varvec{v}= & {} \int _V \int _\Omega \left| \left| \varvec{x} + c\varvec{v}\right| \right| ^2 \rho (t,\varvec{x},\varvec{v}) \text {d}\varvec{x} \text {d}\varvec{v} , \end{aligned}$$

9 $$\begin{aligned}= & {} \int _V \int _\Omega \left( \left| \left| \varvec{x}\right| \right| ^2 + 2c(\varvec{v}\cdot \varvec{x})\right. \nonumber \\&\left. +\,c^2 \left| \left| \varvec{v}\right| \right| ^2 \right) \rho (t,\varvec{x},\varvec{v}) \text {d}\varvec{x} \text {d}\varvec{v} , \end{aligned}$$

10 $$\begin{aligned}= & {} D_\rho ^2(t) + 2c B_\rho (t) + c^2 V_\rho ^2(t) , \end{aligned}$$

and similarly

11 $$\begin{aligned} \int _V \int _\Omega (\varvec{v}\cdot \varvec{x}) \rho (t,\varvec{x}-c\varvec{v},\varvec{v}) \text {d}\varvec{x} \text {d}\varvec{v}= & {} \int _V \int _\Omega (\varvec{v}\cdot \varvec{x} + c\varvec{v}) \rho (t,\varvec{x},\varvec{v}) \text {d}\varvec{x} \text {d}\varvec{v} , \end{aligned}$$

12 $$\begin{aligned}= & {} B_\rho (t) + c V_\rho ^2(t) . \end{aligned}$$

For conservation of mass, $N_p(t) + N_r(t) = N_0$, we see that

13 $$\begin{aligned} \frac{\text {d}N_p(t)}{\text {d}t} = {-} \frac{\text {d}N_r(t)}{\text {d}t} = -\int _0^t \Phi _\tau (t-s)N_p(s)\text {d}s \!+\! \int _0^t \Phi _\omega (t-s)N_r (s) \text {d}s. \end{aligned}$$

Equally, we obtain a system of equations for the MSD

14 $$\begin{aligned} \frac{\text {d}D_p^2(t)}{\text {d}t} - 2B_p(t)= & {} -\int _0^t \Phi _\tau (t-s)\left[ D_p^2(s) + 2(t-s)B_p(s) + (t{-}s)^2V_p^2(s)\right] \text {d}s, \nonumber \\&+ \int _0^t \Phi _\omega (t{-}s) D_r^2(s) \text {d}s = - \frac{\text {d}D_r^2(t)}{\text {d}t}. \end{aligned}$$

For the mean velocity-displacement, we see that

15 $$\begin{aligned} \frac{\text {d}B_p(t)}{\text {d}t}= & {} V_p^2(t) - \int _0^t \Phi _\tau (t-s) \left[ B_p(s) + (t-s)V_p^2(s)\right] \text {d}s, \\&+\,\psi _d \int _0^t \Phi _\omega (t-s) B_r(s) \text {d}s, \nonumber \end{aligned}$$

and

16 $$\begin{aligned} \frac{\text {d}B_r(t) }{\text {d}t} = - \int _0^t \Phi _\omega (t-s) B_r(s) \text {d}s+ \int _0^t \Phi _\tau (t-s) \left[ B_p(s) + (t-s)V_p^2(s)\right] \text {d}s. \end{aligned}$$

Finally, for the second velocity moment:

17 $$\begin{aligned} \frac{\text {d}V_p^2(t)}{\text {d}t}= & {} - \int _0^t \Phi _\tau (t-s) V_p^2(s) \text {d}s +S_T^2 \int _0^t \Phi _\omega (t-s) N_r(s) \text {d}s, \end{aligned}$$

18 $$\begin{aligned} \frac{\text {d}V_r^2(t)}{\text {d}t}= & {} - \int _0^t \Phi _\omega (t-s) V_r^2(s) \text {d}s+ \int _0^t \Phi _\tau (t-s) V_p^2 (s) \text {d}s. \end{aligned}$$

Equations (13–18) above correspond to a system of 8 equations, or 7 unique equations once we impose conservation of mass. In the next section, we solve these equations numerically, the integrals are calculated using the trapezoidal rule and a Crank–Nicholson scheme is applied for the remaining differential operators. Both of these methods are second-order accurate.

## Comparison Between Theory and Experiment

In this study, we consider experimental data relating to the bacterium *E. coli* and the lesser black-backed gull *L. fuscus*. Both of these exhibit somewhat similar behaviour, however, at scales many orders of magnitude apart.

### *E. coli*

There is a large collection of work relating to studying the run-and-tumble motion as exhibited in many flagellated bacteria (Berg and Brown 1972; Frymier et al. 1995; Rosser et al. 2013; Wu et al. 2006). A case of particular interest to many is *E. coli*, perhaps due to the fact that its internal signalling pathways are less complex than those of other chemotactic bacteria (Porter et al. 2008). Most available literature points to both the running and resting times being exponentially distributed (Berg 1990). The rate parameter changes adaptively in response to the surrounding environment, giving rise to the phenomenon of chemotaxis either towards nutrients or away from toxins (Erban and Othmer 2004, 2005).

In our case, however, we consider *E. coli* swimming freely in the absence of any chemical gradient. The data set used here has previously been described in studies by Rosser et al. (2013, 2014). In brief, the data were obtained by performing video microscopy on samples of free-swimming *E. coli*, from which tracks were extracted using a kernel-based filter (Wood et al. 2012). The tracks were subsequently analysed using a hidden Markov model (HMM) to infer the state (running or resting) attributed to the motion between each pair of observations in a track (Rosser et al. 2013). From the annotated tracks, it is possible to extract the angle changes observed between running phases and parameters for the exponential running and waiting pdfs along with speed distributions.

#### Results

**Fig. 1:**
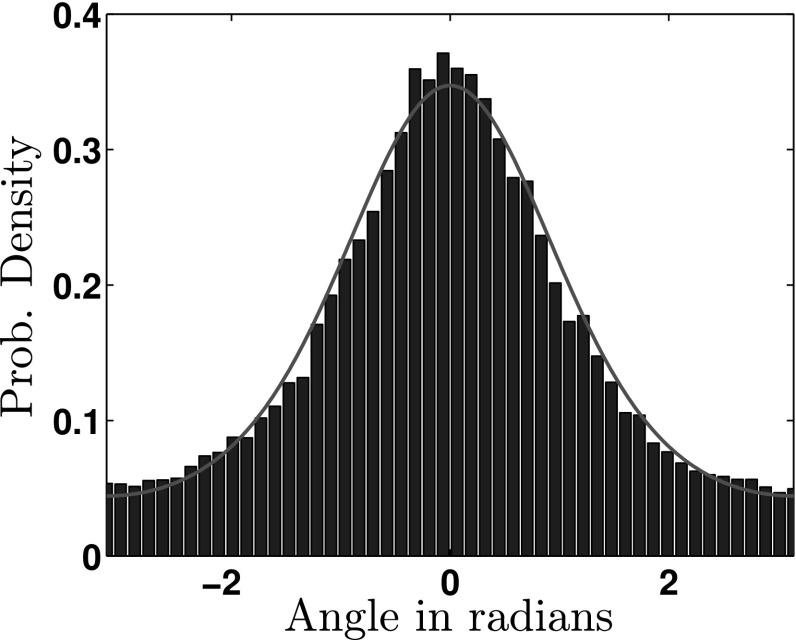
Fit between experimentally observed values of angle changes from run-to-run (*blue*) and the probability density function for the von Mises distribution (*red*) (Color figure online)

In Fig. 1, we see that from run-to-run, the distribution of angles is well described by the von Mises distribution (red line), which is a close approximation to the wrapped normal distribution with a more analytically tractable functional form. The von Mises pdf is given by

19 $$\begin{aligned} \Theta (\theta | \mu , \kappa ) = \frac{e^{\kappa \cos (\theta - \mu )}}{2\pi I_0(\kappa )}, \quad \text {for }\kappa > 0, \mu \in {\mathbb {R}}, \end{aligned}$$

where $\kappa$ controls the width of the distribution, $\mu$ denotes the mean angle and $I_0(\cdot )$ is the modified Bessel function of order zero. By assuming $g(\theta , \theta ') = \Theta (\theta - \theta ')$, i.e. symmetry around the previous direction, we can specify $\mu = 0$ and find $\kappa$ through maximum likelihood estimation. It has been shown that for a two-dimensional von Mises distribution ($n=2$) the index of persistence is given by $\psi _d = I_1(\kappa ) / I_0 (\kappa )$. We find that $\psi _d = 0.46$ for our *E. coli* data set.

It should be noted that from the literature, *E. coli* is thought to have a bi-modal distribution around the previous direction (Berg and Brown 1972), the validity of this is hard to confirm as previous data were hand-annotated, and it is challenging to specify the state of the bacterium when diffusion effects are also in place. Whilst we had more data available to us and used automated tracking methods, it could well be that our method heavily biases walks towards normally distributed reorientation.

**Fig. 2:**
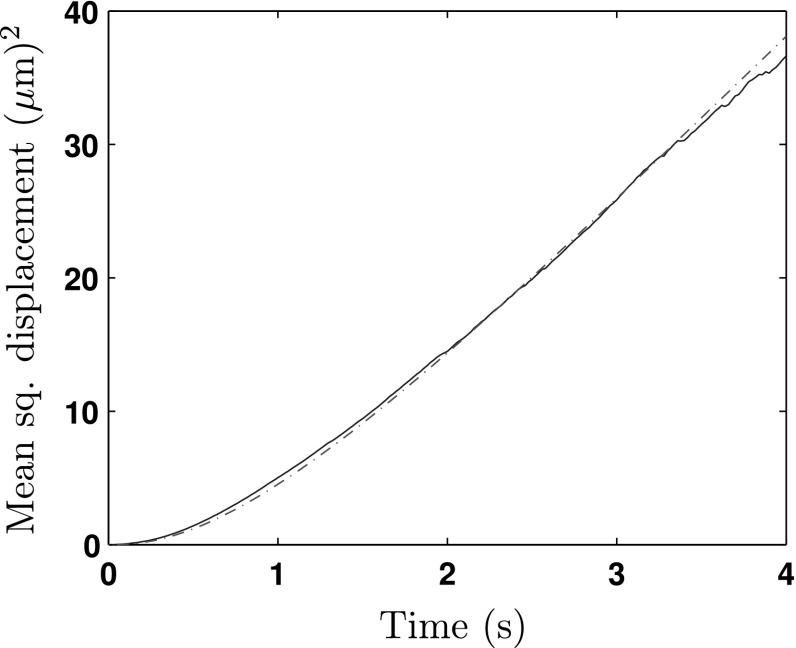
Comparison between system of equations (13–18) and *E. coli* data. The *red dot-dashed line* shows the theoretical value of $(D_p^2 + D_r^2)/N_0$ and the *blue solid line* indicates the experimentally derived average MSD calculated from the bacterium’s initial position. From the data, we determined that $\tau \sim \text {Exp}(2.30), \omega \sim \text {Exp}(11.98)$. For the system of differential equations, we specify $N_p(0) = 66, N_r(0) = 1802, \psi _d = 0.46$ and $S_T^2 = 9.26\,(\mu \text {m})^2/\text {s}$. The initial state for all other differential equations is set to zero, except for $V_p^2(0) = S_T^2 N_p^2(0)$ (Color figure online)

Through the HMM analysis technique outlined in Rosser et al. (2013, 2014), estimates for the exponential parameters were found to be $\tau \sim \text {Exp}(2.30)$ and $\omega \sim \text {Exp}(11.98)$. The mean squared speed whilst running was calculated to be $S_T^2 = 9.26\,(\mu \text {m})^2/\text {s}$. In Fig. 2, we plot the MSD over time. Initial conditions $N_p(0),\, N_r(0)$ were found by counting up the number of *E. coli* labelled to be in each state when $t=0$. We clearly see that over the average of 1868 paths, we get a very good match between theory and experiment. We note that the videos were taken from a fixed position, where bacteria would swim in and out of the shot. By considering the average speeds of *E. coli* along with the size of the viewing window, one can stipulate that by only considering the MSD before 4 s, we can achieve a good estimate. Note that we lose a small amount of data over time as bacterium swim out of the observation window, at later times this ruins the validity of the MSD curve.

### Lesser Black-Backed Gull

**Fig. 3:**
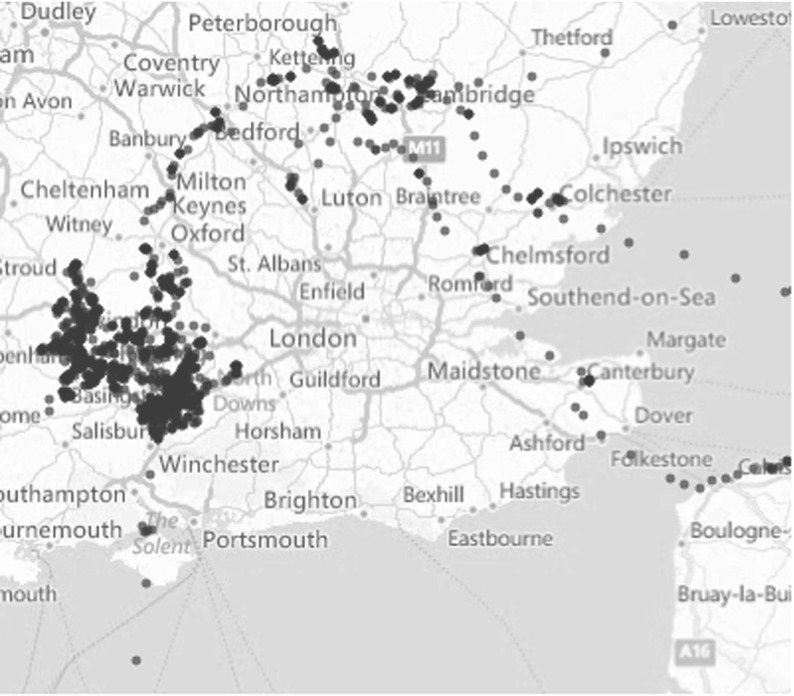
Sample path from bird data (Color figure online)

In this section, we consider Lesser black-backed gulls that breed on Texel (the Netherlands). During their non-breeding period (August–April), these birds interchange between localised movements (or resting) and long-distance movements (migration) (Bouten et al. 2013; Klaassen et al. 2012). During the resting mode, birds travel up to 50 km but return to a central place every day, whereas during the migration mode birds do not return to the central place and can travel several hundreds of kilometres per day. One point of interest is that whilst the resting periods can last months on end, the migrations may only last for a few days on end. See Fig. 3 for a section of a sample path centred around London.

#### Identification of States

The bird tracking data were collected by the UvA-BiTS system (Bouten et al. 2013) and contain tracks gathered from 10 birds over the months July until January in the years 2012 and 2013. Approximately every few hours,^3^ a recording is taken of a global time stamp along with the bird’s current latitude and longitude coordinates.

To identify the state of a given bird, we create a signal centred around a time point of interest which we threshold to determine whether the bird is either undergoing local or migratory behaviour. By considering all GPS coordinates in a 24 h window, we calculate the diameter of the convex hull^4^ (or diameter of a minimum bounding circle) of the set by using the Haversine formula.^5^ This signal is sampled 10 times a day. If the value of this signal is low, points are clustered together (local resting behaviour); otherwise, they are spread apart (migratory behaviour). At the cost of including some erroneous exceptionally short rests, we can set a low threshold value of 52 km; the presence of short rests is then fixed by discarding any resting phases shorter than 2 days. In comparison, the running periods can virtually be of any length as there have been instances of a bird flying exceptionally long distances over a week.

In Fig. 3, the trajectories plotted appear curved in places, with the apparent curvature persisting for short distances. The process of sampling from the trajectory ten times per day removes any such fine-scale effects. In addition to reducing the noise in the data (in the form of GPS tracking ‘jitter’), the process of downsampling tracks makes the result more amenable to modelling with the VJ framework. A caveat of this approach is that angle changes between trajectory segments will not be measured to a high degree of accuracy (a discussion of this issue is found in Rosser et al. 2013). Sensitivity analysis, showing changes to our predicted MSD with respect to changes in parameters is given in the supplementary material.

#### Results

As we only had the data for 10 birds available, we divided their sample paths up into 28-day intervals after approximating distributions of interest, leading to calculation of the MSD over 62 sample paths. In contrast to the *E. coli* data set, we see that running and waiting times are not exponentially distributed. The distribution of running and waiting times was approximated by inverse Gaussian distributions $\tau \sim \text {IG}(1.26,1.22)$ and $\omega \sim \text {IG}(10.79,7.42)$; these distributions are highly flexible and are a convenient choice numerically as they have an exact analytic Laplace transform. The speed distribution gave an estimate for the mean squared running speed as $S_T^2 = 1.03\times 10^{5}\, (\text {km})^2 / \text {day}$ and, again using a von Mises angular distribution, we find $\psi _d = 0.42$. As before, initial conditions $N_p(0),\, N_r(0)$ were found by counting up the number of *L. fuscus* labelled to be in each state when $t=0$.

**Fig. 4:**
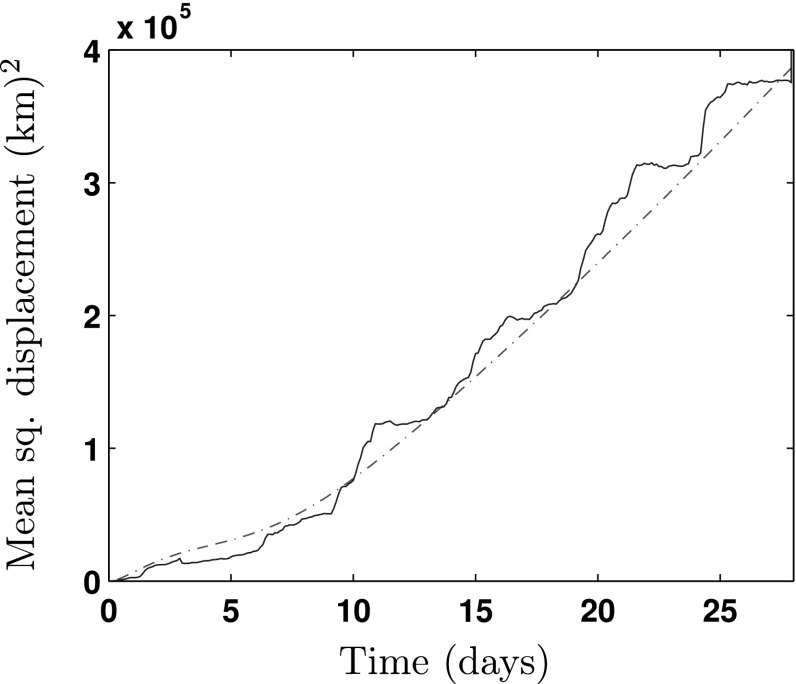
Comparison between system of Eqs. (13–18) and *Larus fuscus* data. The *red dot-dashed line* shows the theoretical value of $(D_p^2 + D_r^2)/N_0$ and the *blue solid line* indicates the experimentally derived average MSD calculated from the bird’s initial position. From the data, it was extracted that $\tau \sim \text {IG}(1.26,1.22), \omega \sim \text {IG}(10.79,7.42)$. For the system of differential equations, we specify $N_p(0) = 6, N_r(0) = 56, \psi _d = 0.42$ and $S_T^2 = 1.03\times 10^{5}\, (\text {km})^2 / \text {day}$. The initial state for all other differential equations is set to zero, except for $V_p^2(0) = S_T^2 N_p^2(0)$ (Color figure online)

In Fig. 4, we plot the MSD in kilometres squared against time in days. As there were fewer sample paths available, the empirical MSD curve is not very smooth, and as a result, the agreement with the theoretical curve is less good than in the bacterial case. However as the majority of the gulls were in a resting state to begin with, we do capture the initial delay before a linear growth stage. As the gulls are frequently resting as opposed to migrating, we see the data for the gulls (in blue) undergoing a style of step function where a small number of gulls undergoing fast movement quickly changes the MSD for the whole population. As the number of sample paths increases, this effect will smooth out.

### Finishing Remarks

At time $t=0$, we need to give the numbers of particles in the running and resting phases, respectively. To find these initial conditions from experimental data, our sample paths had their state assigned to them (running or resting) for all times *t* over which the sample path was valid. We then looked at the beginning of each sample path and chose the initial state to be the state at $t=0$ regardless of whether the particle had just started a run or rest, or was midway through one.

We note that the assumption that particles have just started a run or rest at $t=0$ introduces errors in the estimation of $f_\tau (t)$ and $f_\omega (t)$, since the duration of the first phase is systematically underestimated with this approach. However, the errors are expected to be small as relatively few erroneous data are introduced by this means. A quantification of the error could be carried out systematically with a simulation study, but this is beyond the scope of this paper. If tracks of a longer duration are available, one may instead truncate the beginning of each track up to the beginning of the first running/resting phase.

Finally, we note that, when dealing with integro-differential equations in time, it is maybe natural to specify an initial history function, rather than an initial condition. In our case, this would correspond to the number of particles having just started a run or a rest for all times $t<0$; numerically, this is impractical.

With both examples, as time passes, we see that $D_p^2(t) + D_r^2(t) \sim t$ for large *t*. It is well known that linear MSD corresponds to the solution of the diffusion equation, or at least diffusive-like behaviour. This motivates us to seek a diffusion approximation for large time in Sect. 5. Othmer demonstrated that, for exponentially distributed running and waiting times, the small time behaviour of the MSD is quadratic before relaxing to linear behaviour (Othmer et al. 1988). This is indeed the behaviour observed in Fig. 2 for *E. coli*. However, the small time behaviour is not yet known for general waiting kernels. Whilst this is beyond the scope of the present work, we note from Fig. 4 that the behaviour is more complex in the case of *L. fuscus*.

For position jump processes with repositioning/waiting kernels with infinite higher-order moments (i.e. Lévy flights), fractional diffusion equations are required to model the behaviour of sample paths (Metzler and Klafter 2000). In the case where running and waiting distributions do not have finite moments, we expect there to be a large time asymptotic regime where our VJ equations can be approximated via a fractional diffusion equation.

## Large Time Diffusion Approximation

We now construct a large time effective diffusion equation. By first considering Eqs. (1–2), we transform into Laplace space, where large values of *t* correspond to small values of the Laplace variable $\lambda$. We then carry out a Taylor expansion of the delay kernels to remove the convolutions in time (see Eqs. 54–55 in Appendix 1 for details).

Converting back to the time domain, one obtains

20 $$\begin{aligned} (1 + \bar{\Phi }_\tau '(0))\left( \frac{\partial }{\partial t} + \varvec{v}\cdot {\nabla _{\varvec{x}}} \right) p= & {} -\bar{\Phi }_\tau (0) p + \int _V T(\varvec{v},\varvec{v}')\left( \bar{\Phi }_\omega (0) r(t,\varvec{x},\varvec{v}') \right. \nonumber \\&\left. +\,\bar{\Phi }_\omega '(0)\frac{\partial }{\partial t} r(t,\varvec{x},\varvec{v}') \right) \text {d}\varvec{v}' , \end{aligned}$$

and

21 $$\begin{aligned} (1 + \bar{\Phi }_\omega '(0))\frac{\partial }{\partial t}r = -\bar{\Phi }_\omega (0) r +\bar{\Phi }_\tau (0) p + \bar{\Phi }_\tau '(0)\left( \frac{\partial }{\partial t} + \varvec{v}\cdot {\nabla _{\varvec{x}}} \right) p \end{aligned}$$

There are now two further steps to obtain an effective diffusion equation. First, by considering successively greater monomial moments in the velocity space, one obtains a system of *k*-equations where the equation for the time evolution of moment *k* corresponds to the flux of moment $k+1$. It therefore becomes necessary to ‘close’ the system of equations to create something mathematically tractable. We use the Cattaneo approximation for this purpose (Hillen 2003, 2004). Once a closed system of equations has been found, we then carry out an asymptotic expansion where we investigate the parabolic regime to obtain a single equation for the evolution of the density of particles at large time.

Note that it would be possible to carry out a similar process for smaller time behaviour by Taylor expanding the spatial delays in the convolution integrals. Asymptotic analysis would then have to be carried out to simplify the remaining convolution.

### Moment Equations

We can multiply Eqs. (20–21) by monomials in $\varvec{v}$ and integrate over the velocity space to obtain equations for the velocity moments

22 $$\begin{aligned} m^0_\rho = \int _V \rho (t,\varvec{x},\varvec{v})\text {d}\varvec{v}, \quad \varvec{m}^1_\rho = \int _V \varvec{v}\rho (t,\varvec{x},\varvec{v})\text {d}\varvec{v}, \quad M^2_\rho = \int _V \varvec{v}\varvec{v}^T\rho (t,\varvec{x},\varvec{v})\text {d}\varvec{v}. \end{aligned}$$

The equations relating the terms $m_p^0, m_r^0, \varvec{m}_p^1, \varvec{m}_r^1, M_p^2$ are given below. For initial integration over the velocity space, we see

23 $$\begin{aligned} (1 + \bar{\Phi }_\tau '(0))\left( \frac{\partial m^0_p}{\partial t} + \nabla _{\varvec{x}} \cdot \varvec{m}_p^1 \right) = - \bar{\Phi }_\tau (0)m_p^0 + \bar{\Phi }_\omega (0)m_r^0 + \bar{\Phi }_\omega '(0)\frac{\partial m_r^0}{\partial t} , \end{aligned}$$

and

24 $$\begin{aligned} (1 + \bar{\Phi }_\omega '(0))\frac{\partial m^0_r}{\partial t} = - \bar{\Phi }_\omega (0)m_r^0 + \bar{\Phi }_\tau (0)m_p^0 + \bar{\Phi }_\tau '(0)\left( \frac{\partial m^0_p}{\partial t} + \nabla _{\varvec{x}} \cdot \varvec{m}_p^1 \right) , \end{aligned}$$

When summing Eqs. (23) and (24), we see that mass flux is caused by the movement of particles in the running state only, i.e.

$$\begin{aligned} \frac{\partial }{\partial t}\left( m_p^0 + m_r^0 \right) + \nabla _{\varvec{x}} \cdot \varvec{m}_p^1 = 0. \end{aligned}$$

For multiplication by $\varvec{v}$ and integrating, we obtain equations

25 $$\begin{aligned} (1 + \bar{\Phi }_\tau '(0))\left( \frac{\partial \varvec{m}^1_p}{\partial t} + \nabla _{\varvec{x}} \cdot M_p^2 \right) = - \bar{\Phi }_\tau (0)\varvec{m}_p^1 +\,\psi _d \bar{\Phi }_\omega (0)\varvec{m}_r^1 + \psi _d \bar{\Phi }_\omega '(0)\frac{\partial \varvec{m}_r^1}{\partial t},\nonumber \\ \end{aligned}$$

and

26 $$\begin{aligned}&(1 + \bar{\Phi }_\omega '(0))\frac{\partial \varvec{m}^1_r}{\partial t} = - \bar{\Phi }_\omega (0)\varvec{m}_r^1 + \bar{\Phi }_\tau (0)\varvec{m}_p^1 + \bar{\Phi }_\tau '(0)\left( \frac{\partial \varvec{m}^1_p}{\partial t} + \nabla _{\varvec{x}}\cdot M_p^2 \right) . \end{aligned}$$

We would now like to approximate the $M_p^2$ term to close the system.

### Cattaneo Approximation Step

We make use of the Cattaneo approximation to the VJ equation as studied by Hillen (2003, 2004). For the case where the speed distribution is independent of the previous running step, i.e. $h(s,s') = h(s)$, we approximate $M_p^2$ by the second moment of some function $u_\text {min} = u_\text {min} (t,\varvec{x},\varvec{v})$, such that $u_\text {min}$ has the same first two moments as $p = p(t, \varvec{x}, \varvec{v})$ and is minimised in the $L^2(V)$ norm weighted by $h(s)/s^{n-1}$. This is essentially minimising oscillations in the velocity space whilst simultaneously weighting down speeds which would be unlikely to occur (Hillen 2003).

We introduce Lagrangian multipliers $\Lambda ^0 = \Lambda ^0(t, \varvec{x})$ and $\varvec{\Lambda }^1 =\varvec{\Lambda }^1(t, \varvec{x})$ and then define

27 $$\begin{aligned} H(u) := \frac{1}{2} \int _V \frac{u^2}{h(s)/s^{n-1}}\text {d}\varvec{v} {-} \Lambda ^0\left( \int _V u \text {d}\varvec{v} {-} m_p^0\right) {-} \varvec{\Lambda }^1\cdot \left( \int _V\varvec{v} u \text {d}\varvec{v} - \varvec{m}_p^1\right) . \end{aligned}$$

By the Euler–Lagrange equation (Gregory 2006), we can minimise *H*(*u*) to find that

28 $$\begin{aligned} u(t, \varvec{x}, \varvec{v}) = \frac{\Lambda ^0(t, \varvec{x}) h(s)}{s^{n-1}} + \frac{(\varvec{\Lambda }^1(t, \varvec{x}) \cdot \varvec{v}) h(s)}{s^{n-1}}. \end{aligned}$$

We now use the constraints to find $\Lambda ^0$ and $\varvec{\Lambda }^1$. For $m_p^0$, we have

29 $$\begin{aligned} m_p^0 = \int _V u \text {d}\varvec{v} = \Lambda ^0 \int _V h(s)/s^{n-1} \text {d}\varvec{v} = \Lambda ^0 \text {Area}({\mathbb {S}}^{n-1}), \end{aligned}$$

where  is the *n*-sphere centred at the origin. Notice also that the $\int _V \varvec{v}h(s)/s^{n-1} \text {d}\varvec{v} = \varvec{0}$ by symmetry. For the first moment, we calculate

30 $$\begin{aligned} \varvec{m}_p^1 = \int _V \varvec{v} u \text {d}\varvec{v} = \varvec{\Lambda }^1 \cdot \int _V \varvec{v}\varvec{v}^T h(s)/s^{n-1} \text {d}\varvec{v} = S^2_T \text {Vol}({\mathbb {V}}^{n})\varvec{\Lambda }^1, \end{aligned}$$

where ${\mathbb {V}}^n$ is the closure of , i.e. the ball around the origin. Therefore, we can stipulate the form for $u_{\text {min}}$ as

31 $$\begin{aligned} u_{\text {min}}(t, \varvec{x}, \varvec{v}) = \frac{m_p^0(t, \varvec{x}) h(s)}{s^{n-1}\text {Area}({\mathbb {S}}^{n-1})} + \frac{(\varvec{m}_p^1(t, \varvec{x}) \cdot \varvec{v}) h(s)}{S_T^2s^{n-1}\text {Vol}({\mathbb {V}}^n)}. \end{aligned}$$

We now approximate the second moment of *p* by the second moment of $u_{\text {min}}$.

32 $$\begin{aligned} M^2(u_{\text {min}}) = \int _V \varvec{v}\varvec{v}^T u_{\text {min}}(t,\varvec{x},\varvec{v})\text {d}\varvec{v} = S_T^2 \frac{\text {Vol}({\mathbb {V}}^n)}{\text {Area}({\mathbb {S}}^{n-1})} I_n m_p^0(t, \varvec{x}) = \frac{S_T^2}{n}I_n m_p^0(t, \varvec{x}) . \end{aligned}$$

So in the above equations, we simply approximate $\nabla _{\varvec{x}} \cdot M_p^2 \approx \frac{S_T^2}{n}\nabla _{\varvec{x}}m_p^0$.

### Effective Diffusion Constant

Finally, we rescale our equations using the parabolic regime (Erban and Othmer 2004)

33 $$\begin{aligned} t = \hat{t}/\varepsilon ^2, \quad \varvec{x} = \hat{\varvec{x}} /\varepsilon , \end{aligned}$$

for arbitrary small parameter $\varepsilon > 0$. By putting our variables into vectors $\varvec{u} = (m_p^0, m_r^0)^T$ and $\varvec{v}=(\varvec{m}_p^1,\varvec{m}_r^1)^T$, we drop the hats over the rescaled variables and rewrite our equations as

34 $$\begin{aligned} \varepsilon ^2 \frac{\partial }{\partial t} A\varvec{u} + \varepsilon F \nabla _{\varvec{x}}\cdot \varvec{v} = C\varvec{u}, \quad \varepsilon ^2 \frac{\partial }{\partial t} B\varvec{v} + \varepsilon \frac{S_T^2}{n}F \nabla _{\varvec{x}} \varvec{u} = D\varvec{v}, \end{aligned}$$

where $\nabla _{\varvec{x}} \varvec{u} = [\nabla _{\varvec{x}}m_p^0,\nabla _{\varvec{x}}m_p^0]^T$ and $\nabla _{\varvec{x}} \cdot \varvec{v} = [\nabla _{\varvec{x}}\cdot \varvec{m}_p^1,\nabla _{\varvec{x}}\cdot \varvec{m}_p^1]^T$. Our time derivative matrices are given by

35 $$\begin{aligned} A = \left[ \begin{array}{cc} 1 + \bar{\Phi }_\tau '(0) &{} - \bar{\Phi }_\omega '(0) \\ - \bar{\Phi }_\tau '(0) &{} 1 + \bar{\Phi }_\omega '(0) \\ \end{array} \right] , \quad B = \left[ \begin{array}{cc} 1 + \bar{\Phi }_\tau '(0) &{} - \psi _d\bar{\Phi }_\omega '(0) \\ - \bar{\Phi }_\tau '(0) &{} 1 + \bar{\Phi }_\omega '(0) \\ \end{array} \right] , \end{aligned}$$

our flux matrix is given as

36 $$\begin{aligned} F = \left[ \begin{array}{cc} 1 + \bar{\Phi }_\tau '(0) &{} 0 \\ - \bar{\Phi }_\tau '(0) &{} 0 \\ \end{array} \right] . \end{aligned}$$

Finally, our source terms are

37 $$\begin{aligned} C = \left[ \begin{array}{cc} - \bar{\Phi }_\tau (0) &{} \bar{\Phi }_\omega (0) \\ \bar{\Phi }_\tau (0) &{} -\bar{\Phi }_\omega (0) \\ \end{array}\right] , \quad D = \left[ \begin{array}{cc} - \bar{\Phi }_\tau (0) &{} \psi _d \bar{\Phi }_\omega (0) \\ \bar{\Phi }_\tau (0) &{} - \bar{\Phi }_\omega (0) \\ \end{array}\right] . \end{aligned}$$

By using the regular asymptotic expansion

38 $$\begin{aligned} \varvec{u} = \varvec{u}^0 + \varepsilon \varvec{u}^1 + \varepsilon ^2 \varvec{u}^2 + \cdots ,\quad \varvec{v} = \varvec{v}^0 + \varepsilon \varvec{v}^1 + \varepsilon ^2 \varvec{v}^2 + \cdots \end{aligned}$$

for $\varvec{u}^j = (m_{p(j)}^0, m_{r(j)}^0)^T$ and $\varvec{v}^j = (\varvec{m}_{p(j)}^1,\varvec{m}_{r(j)}^1)^T$, we obtain the set of equations

39 $$\begin{aligned} \begin{array}{ll} {\varepsilon ^0:} &{} C\varvec{u}^0 = \varvec{0} ,\quad D\varvec{v}^0 = \varvec{0}, \\ {\varepsilon ^1:} &{} F\nabla _{\varvec{x}}\cdot \varvec{v}^0 = C\varvec{u}^1 , \quad F\nabla _{\varvec{x}}\varvec{u}^0 = D\varvec{v}^1,\\ {\varepsilon ^2:} &{} \frac{\partial }{\partial t} A\varvec{u}^0 + F \nabla _{\varvec{x}}\cdot \varvec{v}^1 = C\varvec{u}^2 , \\ &{} \frac{\partial }{\partial t} B\varvec{v}^0 +\frac{S_T^2}{n}F \nabla _{\varvec{x}} \varvec{u}^1 = D\varvec{v}^2 . \end{array} \end{aligned}$$

Providing $\psi _d \not = 1$, solving these in order gives rise to the differential equation for total density $m^0 = m_{p(0)}^0 + m_{r(0)}^0$

40 $$\begin{aligned} \frac{\partial }{\partial t} m^0 = D_{\text {eff}} \nabla _{\varvec{x}}^2 m^0, \end{aligned}$$

for

41 $$\begin{aligned} D_{\text {eff}} = \frac{S_T^2}{n}\frac{1}{\bar{\Phi }_\tau (0)}\frac{\bar{\Phi }_\omega (0)}{\bar{\Phi }_\omega (0) + \bar{\Phi }_\tau (0)} \frac{1 + \bar{\Phi }_\tau ' (0)(1-\psi _d)}{1 - \psi _d}. \end{aligned}$$

We now wish to find the values of $\bar{\Phi }_\tau (0), \bar{\Phi }_\omega (0)$ and $\bar{\Phi }_\tau '(0)$. For probability distributions defined over the positive numbers with pdf *f*(*t*), we see that the Laplace transform can be Taylor expanded as

42 $$\begin{aligned} \bar{f}(\lambda ) = 1 - \langle t \rangle \lambda + \frac{1}{2}\langle t^2\rangle \lambda ^2 - \cdots \end{aligned}$$

for small $\lambda$. Therefore, by putting these terms into the expression $\bar{\Phi }(\lambda )$ given by equation (3), provided that the first two moments are finite, we see that

43 $$\begin{aligned} \bar{\Phi }_i (0)= \lim _{\lambda \rightarrow 0}\bar{\Phi }_i(\lambda ) = \frac{1}{\mu _i}, \quad \bar{\Phi }_i '(0)= \lim _{\lambda \rightarrow 0}\bar{\Phi }_i'(\lambda ) = \frac{1}{2}\left( \frac{\sigma _i^2}{\mu _i^2} - 1\right) ,\quad \text {for } i=\tau , \omega , \end{aligned}$$

for mean $\mu _i$ and variance $\sigma _i^2$ of distribution $i=\tau , \omega$, therefore

44 $$\begin{aligned} D_{\text {eff}}= \frac{S_T^2}{n}\frac{\mu _\tau ^2}{\mu _\tau + \mu _\omega } \left[ \frac{1}{1 - \psi _d} + \frac{1}{2}\left( \frac{\sigma _\tau ^2}{\mu _\tau ^2} - 1\right) \right] . \end{aligned}$$

It is noteworthy that the variance of the running time distribution contributes to the diffusion constant, whilst it is independent of the variance of the waiting time distribution. Therefore, up to a first-order approximation, the diffusion constant is only dependent on the mean of the waiting time distribution. Furthermore, when the running time distribution is exponentially distributed, the correction $\bar{\Phi }_\tau '(0)$ is identically zero. So we can view our diffusion constant as the contribution from the exponential component of the running time distribution, plus an additional (non-Markovian) term for non-exponential running times.

When referring back to the experimental data, it can be seen that by the end of the 4 s, the *E. coli* has entered into the diffusive regime with $D \approx 12.5\,(\mu \text {m})^2 / \text {s}$. The *L. fuscus*, however, is yet to reach this state; we can predict that when it does, the corresponding value of the diffusion constant will be $D \approx 4.7\times 10^4\,(\text {km})^2 / \text {day}$, the solution of the MSD equations for greater time periods suggests that this is true.

### Numerical Example

**Fig. 5:**
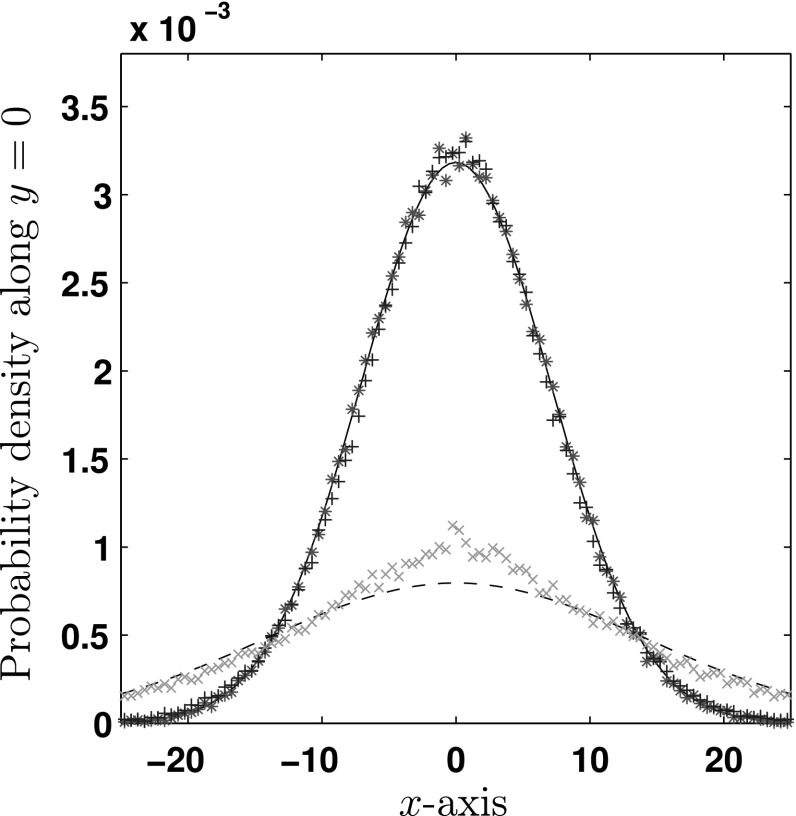
Cross-sectional comparisons along the line $y=0$ between the diffusion equation and Gillespie simulations of the VJ process. In the (*solid*) *black line*, we see the Gaussian solution to the heat equation with diffusion constant $D_{\text {eff}}=1/4$, in the (*dashed*) *black line*, we see the same solution but for diffusion constant $D_{\text {eff}}=1$. For the $D_{\text {eff}}=1/4$ Gillespie simulations, *red asterisks* $(*)$ denote the case where $\tau \sim \text {Exp}(1)$ and $\omega \sim \text {Exp}(1)$, and *blue plusses* $(+)$ show the case $\tau \sim \text {Gamma}(1/5, 5/2)$ and $\omega \sim \text {Exp}(1)$. *Green crosses* ($\times$) show the Gillespie simulation for $\tau \sim \text {Gamma}(1/7, 7)$ and $\omega \sim \text {Gamma}(1/14,14)$ which corresponds to $D_{\text {eff}}=1$ (Color figure online)

We now carry out a comparison between the underlying differential equation and Gillespie simulation. In Fig. 5, we see a comparison between slices of the solution to the diffusion equation on the ${\mathbb {R}}^2$ plane ($n=2$) for a delta function initial condition^6^ compared with data simulated using the algorithm given in Sect. 2.

For the Gillespie simulations, all sample paths are initialised at the origin with fixed speed equal to unity and uniformly random orientation. Therefore, all plots will have the parameters $S_T^2 = 1, \psi _d = 0$, and we specify $\mu _\tau = \mu _\omega = 1$. Plots are shown at $t=100$.

The solid black line shows the solution to the diffusion equation for $D_{\text {eff}} = 1/4$ along the line $y=0$. In red asterisks $(*)$, we see the mean over $3\times 10^5$ Gillespie simulations of the VJ process where both the running and waiting times are sampled from an exponential distribution, with the means of these distributions as stated. This process then has an effective diffusion constant of $D_{\text {eff}} = 1/4$. Using a dashed black line, we plot the solution to the diffusion equation for $D_{\text {eff}} = 1$. In green crosses $(\times )$, a VJ process where the running time is $\tau \sim \text {Gamma}(1/7,7)$ distributed, giving $\mu _\tau = 1$ and $\sigma _\tau ^2 = 7$, the diffusion constant is therefore $D_{\text {eff}} = 1$. The waiting time is $\omega \sim \text {Gamma}(1/14, 14)$ distributed; the high variance of the waiting time is chosen such that the simulation relaxes towards the diffusion approximation quickly. For the above simulations, half the sample paths are initialised in a run and half are initialised in a rest. The gamma and exponential distributions are chosen to illustrate the importance of the non-Markovian term. This is indicated in Fig. 5 by the difference between the two simulations mentioned, which differ only in this correction term.

Another point of interest is that one can model distributions other than exponential with different means and still achieve the same effective diffusion constant through careful selection of variance. An example is shown in Fig. 5 where the diffusion constant $D_{\text {eff}} = 1/4$ is recovered by changing the running distribution to $\tau \sim \text {Gamma}(1/5, 5/2)$ (blue plusses). This then gives a mean run time of $\mu _\tau = 1/2$ and variance $\sigma _\tau ^2 = 5/4$ and compares well to the result for exponentially distributed $\tau$ (red asterisks). For this simulation, 2 / 3 of the sample paths were initialised in a run and the remainder in a resting state so that the system was again encouraged to relax quickly. Viewing these cross sections, one should notice that the fit for the $D_{\text {eff}}=1/4$ case is clearly much better than the fit for $D_{\text {eff}}=1$. Considering equation (44), we suspect that these differences are due to the fact that the running distribution $\tau \sim \text {Gamma}(1/5, 5/2)$ is closer to an exponential distribution, with a smaller non-Markovian contribution to the diffusion constant than the running distribution $\tau \sim \text {Gamma}(1/7, 7)$.

Full heat map figures of the results are given in the supplementary material.

## Discussion and Conclusion

In this study, we have used a single modelling framework to describe two highly distinct biological movement processes, occurring in bacteria and birds. In spite of the significant mechanistic differences between the two species, their phenomenological similarities nonetheless persist over length scales of 10 orders of magnitude. We recover the correct behaviour including the non-local delay effects due to non-exponential waiting times. This formulation could be considered a particularly phenomenological approach as it outlines a way for observables to directly parameterise movement equations. This is counter to some previous literature where quantities such as diffusion constants were left to the reader to identify (McKenzie et al. 2009).

A notable advantage of the modelling framework proposed here is the straightforward interpretation of the distributions and parameters involved, all of which have naturally intuitive meanings. There is, to our knowledge, no unified approach to extract such quantities of interest from biological movement data. This was demonstrated in Sect. 4, in which different approaches were taken to obtain the required parameters. Nonetheless, such methods are the focus of much current research effort (Gautestad et al. 2013; Patterson et al. 2008), and we therefore believe that approaches such as ours will become increasingly relevant in the future.

Furthermore, since we carry out *a priori* estimation of the model parameters by independent analysis, no optimisation routines are required, which avoids introducing concerns about parameter identifiability and overfitting. The very good agreement between theory and experiment demonstrated in Figs. 2 and 4 therefore provides compelling evidence for the applicability of the model developed in this study. A full sensitivity analysis of the model parameters is beyond the scope of this article; however, we demonstrate the effect of independently varying the individual parameters in the supplementary material.

Finally, we demonstrated the novel result that for the underlying stochastic process of interest, the variance of the running time contributes to the large time diffusion constant. This raises the key question: When does the parabolic regime emerge? Our results also act as a warning against using the exponentially distributed running times as an approximation for other distributions, as whilst their mean values may align, the underlying dynamics can change drastically as shown in Fig. 5.

Regarding the accuracy of this generalised VJ framework, it should be realised that the underlying models for the examples given could be improved by making the model more specific to the agent of interest. Below we discuss some possible alterations to the model, which might contribute to greater model realism, albeit whilst incurring a loss of generality.

### Extensions to Model

For *E. coli*, the bacterium is always subject to diffusion; in theory, this should add to its MSD whilst resting and may also affect running phases via rotational diffusion (Rosser et al. 2014). If one wanted to incorporate a small fix to the resting state, it would be simple to add a diffusion term in space to Eq. (2). However, for a more comprehensive solution to the problem, to retain the correlation effects with turning kernel *T*, the Eq. (1) would have a rotational diffusion term added, which is achieved via a Laplacian in the velocity space (Chandrasekhar 1943). Furthermore, equation (2) would have to have to retain its defunct velocity field for orientation in addition to another velocity variable to allow for movement due to diffusion.

For the *L. fuscus*, there are many physical and ecological phenomena, which could to be built into the model; these range from the day–night cycles, in which the bird is reluctant to fly long distances through the night, to geographical effects, where the bird may follow the coastline for navigation. One could also consider environment factors, such as wind influence and availability of food resources. In the work by Chauviere et al. (2010), the authors consider the migration of cells along an extra-cellular matrix. Using a similar formulation to ours, but only considering exponentially distributed waiting times, cells are modelled to preferentially guide themselves along these extra-cellular fibres. A related process is apparent in homing pigeons, which navigate using visible geographical boundaries (Mann et al. 2014). In a similar spirit, the model presented here could be modified so that gulls preferentially align their trajectory with geographical markers.

### Electronic supplementary material

Supplementary material 1 (pdf 1346 KB)

## Appendix 1: Derivation of Two-State Generalised Velocity Jump Process

We can motivate the set of Eqs. (1–2) by considering the temporary variables:

$\eta$: $=\eta (t,\varvec{x},\varvec{v}) :=$ The density of particles at position $\varvec{x}\,\text {d}\varvec{x}$, with velocity $\varvec{v}\,\text {d}\varvec{v}$ at time $t\,\text {d}t$, *having just started a jump*.
$\nu$: $=\nu (t,\varvec{x},\varvec{v}):=$ The density of particles at position $\varvec{x}\,\text {d}\varvec{x}$, *having just finished a jump* of velocity $\varvec{v}\,\text {d}\varvec{v}$ at time $t\,\text {d}t$ and *just started a rest*.

This leads to densities:

*p*: $=p(t,\varvec{x},\varvec{v}):=$ The density of particles at position $\varvec{x}\,\text {d}\varvec{x}$, with velocity $\varvec{v}\,\text {d}\varvec{v}$ at time $t\,\text {d}t$, being in a running state. We should note that we can relate *p* to $\eta$ via the equation 45 $$\begin{aligned} p(t,\varvec{x},\varvec{v})= & {} \int _0^t F_\tau (t-s)\eta (s,\varvec{x}-(t-s)\varvec{v},\varvec{v})\text {d}s \nonumber \\= & {} \int _0^t F_\tau (t-s)e^{-(t-s)\varvec{v}\cdot \nabla _{\varvec{x}}}\eta (s,\varvec{x},\varvec{v})\text {d}s, \end{aligned}$$ for $F_\tau (t) = \int _t^\infty f_\tau (s) \text {d}s$ being the probability that a jump lasts longer than *t*, clearly $F_\tau (0)=1$.
*r*: $=r(t,\varvec{x},\varvec{v}):=$ The density of particles at position $\varvec{x}\,\text {d}\varvec{x}$, having just finishing a jump of velocity $\varvec{v}\,\text {d}\varvec{v}$ at time $t\,\text {d}t$ in a resting state. Equally, there is the relation between *r* and $\nu$, this is 46 $$\begin{aligned} r(t,\varvec{x},\varvec{v}) = \int _0^t F_\omega (t-s)\nu (s,\varvec{x},\varvec{v})\text {d}s, \end{aligned}$$ for $F_\omega (t) = \int _t^\infty f_\omega (s) \text {d}s$ being the probability that a rest lasts longer than *t*, again $F_\omega (0)=1$.

By assuming that at time $t=0$, all particles are initiated into the beginning of a run with distribution $p_0(\varvec{x},\varvec{v})$, we can relate $\eta$ to previous times by the relationship

47 $$\begin{aligned} \eta (t,\varvec{x},\varvec{v}) - p_0(\varvec{x},\varvec{v})\delta (t)= \int _V \int _0^t T(\varvec{v},\varvec{v}')f_\omega (t-s) \nu (s,\varvec{x},\varvec{v}') \text {d}s \text {d}\varvec{v}'. \end{aligned}$$

Again by assuming that particles initiated into the beginning of a rest with distribution $r_0(\varvec{x},\varvec{v})$, there is the recursive relation for $\nu$

48 $$\begin{aligned} \nu (t,\varvec{x},\varvec{v}) - r_0(\varvec{x},\varvec{v})\delta (t)= & {} \int _0^t f_\tau (t-s) \eta (s,\varvec{x}-(t-s)\varvec{v},\varvec{v}) \text {d}s \nonumber \\= & {} \int _0^t f_\tau (t-s) e^{-(t-s)\varvec{v}\cdot \nabla _{\varvec{x}}}\eta (s,\varvec{x},\varvec{v}) \text {d}s. \end{aligned}$$

Taking the Laplace transform in time of Eqs. (45) and (46), we find

49 $$\begin{aligned} \bar{p}(\lambda , \varvec{x}, \varvec{v})= & {} \bar{F}_\tau (\lambda + \varvec{v}\cdot \nabla _{\varvec{x}}) \bar{\eta }(\lambda , \varvec{x}, \varvec{v}), \end{aligned}$$

50 $$\begin{aligned} \bar{r}(\lambda , \varvec{x}, \varvec{v})= & {} \bar{F}_\omega (\lambda )\bar{\nu }(\lambda , \varvec{x}, \varvec{v}). \end{aligned}$$

Equally, taking the Laplace transform of (47) and (48), we see

51 $$\begin{aligned} \bar{\eta }(\lambda , \varvec{x}, \varvec{v}) - p_0(\varvec{x},\varvec{v})= & {} \int _V T(\varvec{v},\varvec{v}') \bar{f}_\omega (\lambda ) \bar{\nu }(\lambda , \varvec{x}, \varvec{v}') \text {d}\varvec{v}', \end{aligned}$$

52 $$\begin{aligned} \bar{\nu }(\lambda , \varvec{x}, \varvec{v}) - r_0(\varvec{x},\varvec{v})= & {} \bar{f}_\tau (\lambda + \varvec{v}\cdot \nabla _{\varvec{x}}) \bar{\eta }(\lambda , \varvec{x}, \varvec{v}). \end{aligned}$$

Noting that in Laplace space

53 $$\begin{aligned} \bar{F}_i(\lambda ) = 1-\bar{f}_i(\lambda )/\lambda , \quad \text {for }i=\tau , \omega , \end{aligned}$$

by eliminating $\eta$ and $\nu$, we derive paired differential equations in Laplace space

54 $$\begin{aligned} \left( \lambda + \varvec{v}\cdot \nabla _{\varvec{x}} \right) \bar{p}(\lambda , \varvec{x},\varvec{v}) - p_0(\varvec{x},\varvec{v})= & {} -\bar{\Phi }_\tau (\lambda + \varvec{v}\cdot \nabla _{\varvec{x}})\bar{p}(\lambda ,\varvec{x},\varvec{v}) \\&+\bar{\Phi }_\omega (\lambda ) \int _V T(\varvec{v},\varvec{v}')\bar{r}(\lambda , \varvec{x},\varvec{v}') \text {d}\varvec{v}', \nonumber \end{aligned}$$

and

55 $$\begin{aligned} \lambda \bar{r}(\lambda , \varvec{x},\varvec{v}) - r_0(\varvec{x},\varvec{v}) = -\bar{\Phi }_\omega (\lambda )\bar{r}(\lambda ,\varvec{x},\varvec{v}) +\bar{\Phi }_\tau (\lambda + \varvec{v}\cdot \nabla _{\varvec{x}}) \bar{p}(\lambda , \varvec{x},\varvec{v}),\nonumber \\ \end{aligned}$$

where, as stated previously^7^

56 $$\begin{aligned} \bar{\Phi }_i (\lambda ) = \frac{\lambda \bar{f}_i (\lambda )}{1 - \bar{f}_i(\lambda )} = \frac{1 - \lambda \bar{F}_i(\lambda )}{\bar{F}_i(\lambda )}, \quad \text {for }i=\tau , \omega . \end{aligned}$$

Reverting back to the temporal variable *t*, we obtain Eqs. (1–2).

## Appendix 2: Properties of Delay Kernel $\Phi$

The integro-differential equations for mean squared displacement, or indeed any other differential equation above, can now be easily solved by a variety of methods for numerical integration. However, in the case where one of the waiting times is exponentially distributed, $\Phi$ has been shown to become a multiple of the delta function at the origin. It can be seen that many other distributions also have a numerical impulse at the origin, numerically integrating over an impulse is often difficult if not impossible so we carry out asymptotic analysis to evaluate the magnitude of said impulse.

To investigate the small time behaviour of $\Phi (t)$, we shall consider the small time behaviour of *f* (*t*) and then transform to Laplace space to consider large $\lambda$ behaviour and subsequently switch back.

By assuming the expansion of *f*(*t*) to be of the form

57 $$\begin{aligned} f(t) \sim f_0 + f_1 t^{\alpha } + \ldots \quad \text {as }t\rightarrow 0. \end{aligned}$$

then subsequently in Laplace space

58 $$\begin{aligned} \bar{f}(\lambda ) \sim \frac{f_0}{\lambda } + \frac{f_1 \Gamma (\alpha + 1)}{\lambda ^{\alpha + 1}} + \ldots \quad \text {for } \alpha > -1,\text { as }\lambda \rightarrow \infty . \end{aligned}$$

Using the relation (3) and considering the minimal contribution of the denominator, we find

59 $$\begin{aligned} \bar{\Phi }(\lambda ) \sim f_0 + \frac{f_1 \Gamma (\alpha + 1)}{\lambda ^\alpha } + \ldots \quad \text {for } \alpha > -1,\text { as }\lambda \rightarrow \infty . \end{aligned}$$

In the case where $\alpha >0$, we can subsequently invert to find

60 $$\begin{aligned} \Phi (t) \sim f_0 \delta (t) + f_1 \alpha t^{\alpha - 1} + \ldots \quad \text {for } \alpha > 0,\text { as } t \rightarrow 0 . \end{aligned}$$

From the above analysis, it should be clear we can expect an impulse at the origin for the case when $f_0 = f(0) \ne 0$ or $f_1\ne 0$ with $\alpha \in (0,1)$. By integrating between 0 and $\varepsilon$, we see that

61 $$\begin{aligned} \int _0^\varepsilon \Phi (t) \text {d}t \sim \int _0^\varepsilon \left[ f_0 \delta (t) + f_1 \alpha t^{\alpha - 1} + \ldots \right] \text {d}t \sim f_0 + f_1 \varepsilon ^\alpha \quad \text {for } \alpha > 0,\text { as } \varepsilon \rightarrow 0 . \end{aligned}$$

## Footnotes

1 The reason for observing diffusive-like behaviour is due to the bacterium’s small size—which is on the length scale of micrometres.

2 This can be seen simply for exponential distribution with mean $\chi ^{-1}$,  where $\delta$ is the Dirac delta function.

3 Although some devices are configured differently to the extent that a recording is taken every 15  min.

4 The maximum distance between any two points in the set.

5 The Haversine formula is an equation for great-circle distances between a pair of points on a sphere. By considering the radius of the sphere (i.e. the approximate radius of the Earth) and a pair of latitude–longitude coordinates, one can calculate the distance between them.

6 This takes the form of a bivariate Gaussian.
