# Supplementary material for: From Birds to Bacteria: Generalised Velocity Jump Processes with Resting States
Source: Bull Math Biol. 2015 Jun 10;77(7):1213–36. doi: 10.1007/s11538-015-0083-7 (PMC4548017; doi:10.1007/s11538-015-0083-7)
Supplement: Supplementary file 1 — Supplementary material 1 (pdf 1346 KB) [file 11538_2015_83_MOESM1_ESM.pdf]

FROM BIRDS TO BACTERIA: GENERALISED VELOCITY JUMP PROCESSES WITH RESTING STATES.

JAKE P. TAYLOR-KING

Supplementary material: Sensitivity analysis of model parameters.

When considering mean squared displacement (MSD), we essentially gave a model that took 4 parameter inputs, S_T^2, ψ_d, $N_p(0)$, $N_r(0)$ and 2 function inputs $f_\tau(t)$, $f_\omega(t)$. We now investigate the effect of varying these inputs. This is important for two reasons. First, it allows us to determine whether varying any single input improves the agreement with the experimental result. Second, it determines which inputs need to be measured to a greater level of accuracy to ensure good agreement.

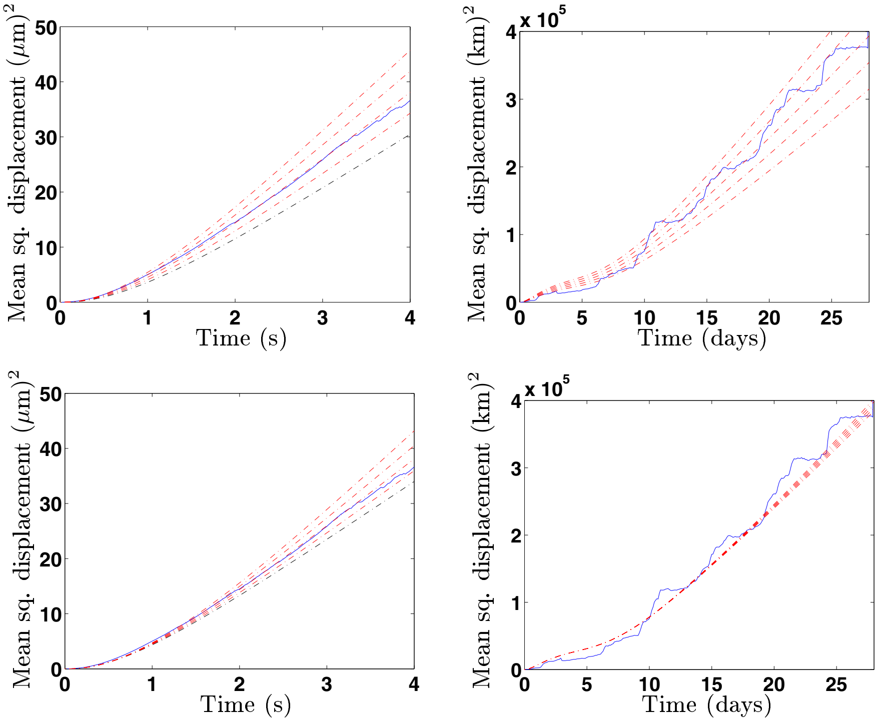

Figure 1. The effect on the MSD of varying the parameters S_T^2 (top row) and ψ_d (bottom row) for *E. coli* (left) and *L. fuscus* (right). All other parameters are as described in the main text. Each parameter is varied between 0.8 and 1.2 of the value measured experimentally, in intervals of 0.1. The black dashed line shows the MSD for a factor of 0.8, red dashed lines show the remaining factors. The experimental result is shown in blue.

Variation of input parameters. In Figure 1, we vary the input parameters S_T^2 and ψ_d by $\pm 20\%$ of the experimentally determined quantity. In Figure 2, we vary the ratio $N_p(0)/(N_p(0)+N_r(0))$ between 0 and 1 using intervals of 0.2.

Varying S_T^2 has the greatest impact for *E. coli*, followed by ψ_d. Unexpectedly, we find that varying the ratio of agents initially in a running phase has minimal impact, despite testing the full permissible range of values.

Date: April 21, 2015.

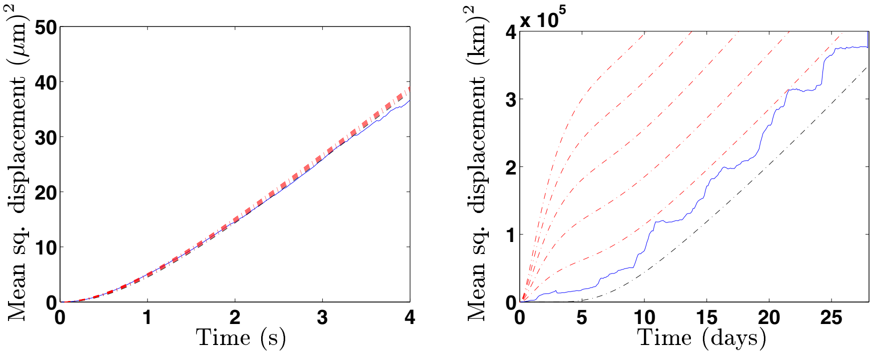

Figure 2. The effect on the MSD of varying the proportion of agents initially in a running state, $N_p(0)/(N_p(0) + N_r(0))$. All other parameters are as described in the main text. The ratio is varied from 0 (black dashed line) to 1 in intervals of 0.2 (red dashed lines). (Left) *E. coli*, (right) *L. fuscus*. The experimental result is shown in blue.

In contrast, variation in initial conditions for *L. fuscus* has the greatest impact. This is in part as we have data over a small timescale when compared to the length of a mean run or rest. Again, S_T^2 has a large impact and ψ_d has minimal effect on the MSD.

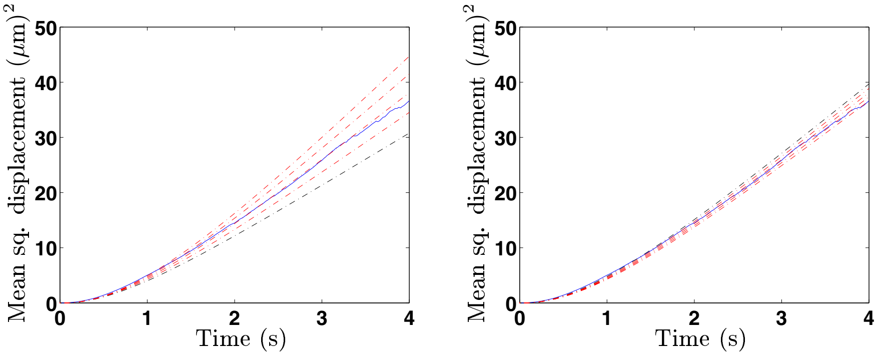

Figure 3. Variation in: (*Left*) the mean of the running time distribution and (*Right*) the mean of the waiting time distribution - for the *E. coli* model. Experimental data plotted in a (solid) blue line and model prediction in a (dot-dashed) red line. As before, varied parameters go between 80%–120% of the measured value, in 10% increments with the 80% valued simulation parameter in black.

Variation of input functions. To vary the input functions, we keep the function form specified for each model, i.e. *E. coli* has exponentially distributed running and waiting times and *L. fuscus* has inverse Gaussian distributed running and waiting times. The exponential distribution is a 1-parameter family of distributions, therefore we vary the mean running and waiting time. The inverse Gaussian distribution is a 2-parameter family of distributions, therefore we first vary the mean, keeping the variance constant, then we vary the variance, keeping the mean constant.

The results for *E. coli* are plotted in Figure 3. Changing the mean run time increases the rate of diffusion, as the bacterium spends most of its time in a running phase. Conversely, as the bacterium stops for small amounts of time varying the mean waiting time has little effect.

The results for *L. fuscus* are plotted in Figure 4. Again, it should be noted that the timescale over which experimental observations are available is not much greater than the mean duration of a run or rest. Therefore small changes in the mean or variance of either distribution will have large impacts on the resulting behaviour. It is noteworthy that lowering the mean run duration actually *increases* the MSD. This outcome is predicted by Equation (5.25) and is due to an increase in the non-Markovian contribution to the effective diffusion coefficient upon lowering the mean run duration.

Supplementary material: Extra plots for numerical example.

Figure 5 shows a comparison between the diffusion approximation (with the effective diffusion coefficient, D_{eff}, defined in Equation (5.25)) and a stochastic simulation of the true underlying movement

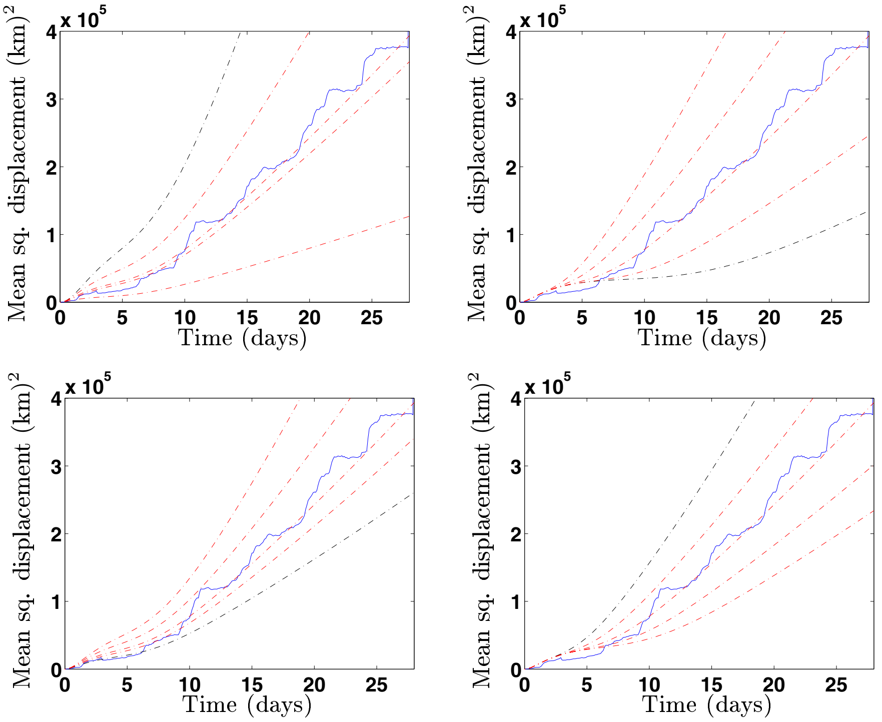

Figure 4. Variation in: (*Top left*) the mean of the running time distribution, (*Top right*) the mean of the waiting time distribution, (*Bottom left*) the variance of the running time distribution and (*Bottom right*) the variance of the waiting time distribution - for the *L. fuscus* model. Experimental data plotted in a (solid) blue line and model prediction in a (dot-dashed) red line. As before, varied parameters go between 80%–120% of the measured value, in 10% increments with the the 80% valued simulation parameter in black.

process. The top row shows results for a VJ process where both the running and waiting times are sampled from an exponential distribution, as is the case for *E. coli.* The mean run and stop duration parameters used here give $D_{\text{eff}} = 1/4$. The bottom row shows a diffusion approximation compared with a VJ process where the running time is $\tau \sim \text{Gamma}(1/7, 7)$ distributed, giving $\mu_\tau = 1$ and $\sigma_\tau^2 = 7$, the diffusion constant is therefore $D_{\text{eff}} = 1$. The waiting time is $\omega \sim \text{Gamma}(1/14, 14)$ distributed.

As before, one can model distributions other than exponential with different means and still achieve the same effective diffusion constant through careful selection of variance. An example is shown in Figure 6, where the diffusion constant $D_{\text{eff}} = 1/4$ is recovered by changing the running distribution to $\tau \sim \text{Gamma}(1/5, 5/2)$.

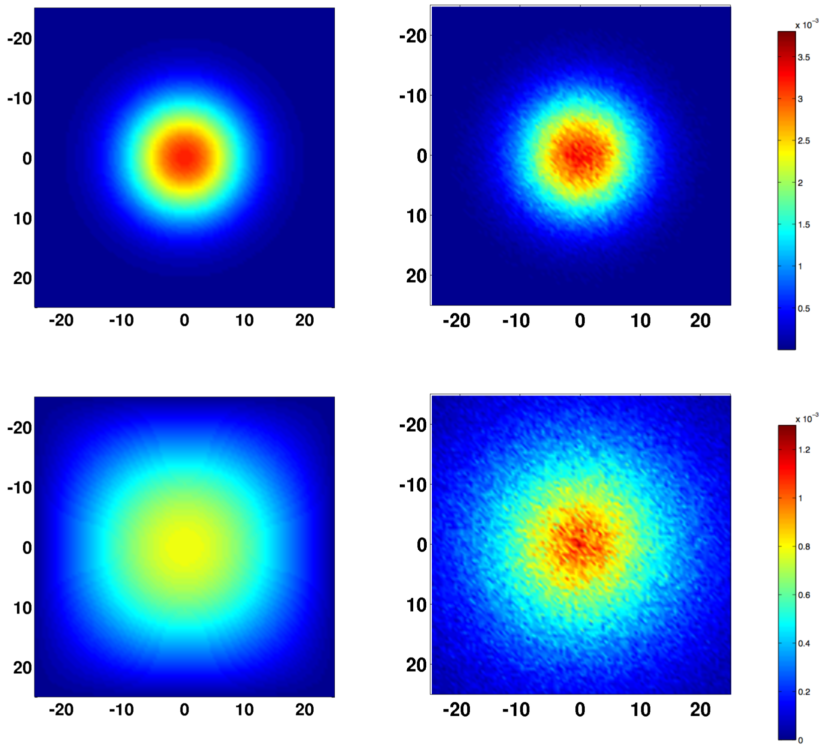

Figure 5. Comparison between solution to the diffusion equation (*left column*) and Gillespie simulation (*right column*). *Top row*: case where $\tau \sim \text{Exp}(1)$ and $\omega \sim \text{Exp}(1)$. *Bottom row*: case where $\tau \sim \text{Gamma}(1/7, 7)$ and $\omega \sim \text{Gamma}(1/14, 14)$. For all Gillespie simulations, 3×10^5 runs were carried out with half initialised in a running phase and half initialised in the resting phase.

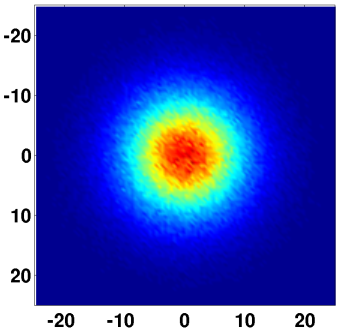

Figure 6. Gillespie simulation for $\tau \sim \text{Gamma}(1/5, 5/2)$ and $\omega \sim \text{Exp}(1)$. For the Gillespie simulation, 2×10^5 simulations were initialised in a running phase and 10^5 were initialised in a resting phase.
